# Adult Female Sleep During Hypoxic Bed Rest

**DOI:** 10.3389/fnins.2022.852741

**Published:** 2022-05-10

**Authors:** Jeroen Van Cutsem, Nathalie Pattyn, Olivier Mairesse, Bérénice Delwiche, Helio Fernandez Tellez, Martine Van Puyvelde, Emilie Lacroix, Adam C. McDonnell, Ola Eiken, Igor B. Mekjavic

**Affiliations:** ^1^VIPER Research Unit, Royal Military Academy, Brussels, Belgium; ^2^Human Physiology and Sports Physiotherapy Research Group, Vrije Universiteit Brussel, Brussels, Belgium; ^3^Sleep Laboratory and Unit for Chronobiology U78, Brugmann University Hospital, Vrije Universiteit Brussel, Brussels, Belgium; ^4^Brain, Body and Cognition, Department of Psychology, Faculty of Psychology and Educational Sciences, Vrije Universiteit Brussel, Brussels, Belgium; ^5^Experimental and Applied Psychology, Department of Psychology and Educational Sciences, Vrije Universiteit Brussel, Brussels, Belgium; ^6^Clinical & Lifespan Psychology, Department of Psychology and Educational Sciences, Vrije Universiteit Brussel, Brussels, Belgium; ^7^Department of Automation, Biocybernetics and Robotics, Jozef Stefan Institute, Ljubljana, Slovenia; ^8^Department of Environmental Physiology, Swedish Aerospace Physiology centre, Royal Institute of Technology, Stockholm, Sweden; ^9^Department of Biomedical Physiology and Kinesiology, Simon Fraser University, Burnaby, BC, Canada

**Keywords:** sleep, polysomnography, altitude, bed rest, female, sex-specific differences

## Abstract

**Purpose:**

Hypobaric hypoxic habitats are currently being touted as a potential solution to minimise decompression procedures in preparation for extra vehicular activities during future space missions. Since astronauts will live in hypoxic environments for the duration of such missions, the present study sought to elucidate the separate and combined effects of inactivity [simulated with the experimental bed rest (BR) model] and hypoxia on sleep characteristics in women.

**Methods:**

Twelve women (Age = 27 ± 3 year) took part in three 10-day interventions, in a repeated measures cross-over counterbalanced design: (1) normobaric normoxic BR (NBR), (2) normobaric hypoxic BR (HBR; simulated altitude of 4,000 m), and (3) normobaric hypoxic ambulatory (HAMB; 4,000 m) confinement, during which sleep was assessed on night 1 and night 10 with polysomnography. In addition, one baseline sleep assessment was performed. This baseline assessment, although lacking a confinement aspect, was included statistically as a fourth comparison (i.e., pseudo normobaric normoxic ambulatory; pNAMB) in the present study.

**Results:**

Hypoxia decreased sleep efficiency (*p* = 0.019), increased N1% sleep (*p* = 0.030), decreased N3 sleep duration (*p* = 0.003), and increased apnea hypopnea index (*p* < 0.001). BR impaired sleep maintenance, efficiency, and architecture [e.g., N2% sleep increased (*p* = 0.033)]. Specifically, for N3% sleep, the effects of partial pressure of oxygen and activity interacted. Hypoxia decreased N3% sleep both when active (pNAMB vs HAMB; *p* < 0.001) and inactive (NBR vs HBR; *p* = 0.021), however, this decrease was attenuated in the inactive state (–3.8%) compared to the active state (–10.2%).

**Conclusion:**

A 10-day exposure to hypoxia and BR negatively impacted sleep on multiple levels as in macrostructure, microstructure and respiratory functioning. Interestingly, hypoxia appeared to have less adverse effects on sleep macrostructure while the participants were inactive (bed ridden) compared to when ambulatory. Data were missing to some extent (i.e., 20.8%). Therefore, multiple imputation was used, and our results should be considered as exploratory.

## Introduction

Maintaining a hypobaric hypoxic environment within a spacecraft or planetary habitat is being considered as a potential solution to minimise decompression procedures in preparation for extravehicular activities (EVAs), and extrahabitat activities on the Moon and Mars (Norcross et al., 2015). The boundary conditions for such future ambients have not yet been resolved. Nevertheless, the equivalent altitudes will most likely be more than 1,500 m (Norcross et al., 2005, 2013), even if the reduced pressure is partly counteracted by increasing the ambient oxygen concentration. This type of environment has a large effect on multiple systems from musculoskeletal and cardiovascular to sleep.

Many normobaric hypoxic (Rojc et al., 2014; Morrison et al., 2017) and hypobaric hypoxic field (Reite et al., 1975; Bloch et al., 1985; Pattyn et al., 1985; Szymczak et al., 2009; Johnson et al., 2010; Stadelmann et al., 2013; Fernandez Tellez et al., 2014; Bian et al., 2019; Mairesse et al., 2019) and laboratory (Anholm et al., 1992; Houston, 1997; Richalet, 2010) studies have focused on the analysis of sleep at altitude, confirming that with increasing altitude there is also an increased level of sleep disturbance. Regarding the combination of hypoxia and bed rest (BR) far less research has been performed (Rojc et al., 2014; Morrison et al., 2017; Gennser et al., 2018). Nevertheless, this specific combination might disrupt sleep even more. BR studies conducted in hypoxia have, for example, indicated that BR augments the detrimental effects of hypoxia on sleep (Rojc et al., 2014; Morrison et al., 2017; Gennser et al., 2018). Morrison et al. (2017) reported that sleep architecture was negatively impacted by both BR and hypoxia and that sleep deteriorated further by combining BR and hypoxia. In contrast to Tellez et al. (2016), Morrison et al. (2017) did not investigate the effect(s) of combining BR and hypoxia on sleep, but their results do, however, add interesting insights on the matter. Tellez et al. (2016) demonstrated that habitual exercise (i.e., the opposite of BR) in hypobaric hypoxic confinement negatively impacts breathing during sleep. Moreover, no acclimatisation was present throughout a period of one year and exercise kept exacerbating sleep-related periodic breathing in hypoxia (Tellez et al., 2016). The contradicting results of Tellez et al. (2016) and Morrison et al. (2017), that both BR and habitual exercise appear to aggravate the hypoxia-related impairments in sleep, calls for more research to investigate the possible interaction between hypoxia and BR.

The aim of the present study was to statistically assess the combined effect of BR and hypoxia on sleep characteristics in women. Although some physiological responses to microgravity and hypoxic conditions exhibit sex-specific differences (Mark et al., 2014), fundamental psychophysiological research in human female cohorts remains relatively sparse (Johnson et al., 2010). We hypothesised that, the hypoxia-induced increase in central and obstructive sleep apnea would negatively impact sleep fragmentation (Ainslie et al., 2013; Fernandez Tellez et al., 2014). We also expected BR to decrease the homeostatic sleep drive, hence decreasing slow-wave sleep and increasing sleep fragmentation (Driver and Taylor, 2000; Rojc et al., 2014; Grønli et al., 2016; Morrison et al., 2017). Lastly, despite previous indications of a reinforcing interaction between the negative effects of BR and hypoxia on sleep (Nespoulet et al., 2012; Morrison et al., 2017), we also hypothesised that BR might attenuate the negative effects of hypoxia on sleep parameters (i.e., an attenuating interaction; Tellez et al., 2016). In addition, a specific focus of this paper was also to assess possible sex-specific differences in the effects of BR and hypoxia on sleep characteristics. To evaluate this, pre-defined hypotheses were put forward based on the findings reported by Rojc et al. (2014) (i.e., the male version of the present study): (1) Hypoxia will reduce the percentage amount of N3 sleep and increase rapid eye movement (REM) sleep, (2) Only when ambulatory, and not in BR, will sleep stage values return to baseline levels after sustained hypoxic exposure (i.e., 10 days), (3) BR will increase the number of awakenings compared to baseline, (4) adaptation to BR will return the number of awakenings to baseline values.

## Materials and Methods

### Ethical Approval

The study conformed to the standards set by the Declaration of Helsinki, except for the registration in a database. The procedures were approved by the Committee for Medical Ethics at the Ministry of Health (Republic of Slovenia; approval number: 88/04/12). Participants were informed regarding the nature of the study and details of the experimental protocol and measurements. Prior to participating in the study, they gave their written informed consent. They were aware that they could terminate their participation and withdraw from the study at any time. The experimental procedures were conducted according to the ESA recommendations (Heer et al., 2009).

### Participants

The inclusion/exclusion criteria were based on ESAs’ recommendations for BR protocols (Heer et al., 2009). Individuals exposed to altitudes above 2,000 m within 2 months prior to the start of the study were ineligible to participate. All participants provided informed consent and were given detailed information regarding the research protocol and all experimental procedures. Twelve participants were included in the final analysis (age = 27 ± 3 year; height = 167 ± 5 cm; weight = 59 ± 8 kg; body mass index = 21 ± 2 kg m^–1^; body fat = 29 ± 5%; V̇O_2_ = 40 ± 4 ml kg^–1^ min^–1^).

### Experimental Protocol

**FIGURE 1:**
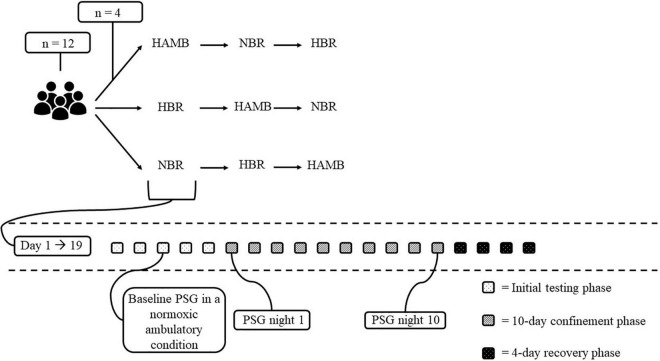
Overview of the experimental design. HAMB, normobaric hypoxic ambulatory; NBR, normobaric normoxic BR; HBR, normobaric hypoxic BR. The baseline PSG in a normobaric normoxic ambulatory condition (i.e., pNAMB) was performed once in the initial testing phase of one of the three conditions (i.e., HAMB, NBR, or HBR). For all participants the baseline PSG recording was performed before the HAMB-condition.

The present study was part of the FemHab (Planetary Habitat Simulation with female participants) project supported in part by ESA. The overall goal of the project was to assess the process of adaptation to inactivity and unloading of the weight bearing limbs in different physiological systems, and compare this to the adaptations observed in similar studies in men, specifically with the results of the LunHab study (Rojc et al., 2014; McDonnell et al., 2019). The environmental conditions within the facility where the interventions were performed were stable throughout (ambient temperature = 24.6 ± 0.6°C, relative humidity = 42 ± 5%, and ambient pressure = 684 ± 5 mmHg). This prospective, randomised, and placebo-controlled trial, was performed at the Olympic Sport Centre Planica (a European Space Agency ground-based research facility in Planica-Rateče, Slovenia), located at an altitude of 940 m. Participants were invited to participate in a 3-arm cross-over design study comprising the following 10-day interventions: (1) normobaric normoxic BR [NBR; fraction of inspired O_2_ (F_I_O_2_) = 0.209; partial pressure of inspired O_2_ (P_*I*_O_2_) 132.9 ± 0.3 mmHg], (2) normobaric hypoxic BR [HBR; F_I_O_2_ = 0.142 ± 0.001; P_*I*_O_2_ 90.4 ± 0.3 mmHg; 4,000 m simulated altitude (Reite et al., 1975)], and (3) normobaric hypoxic ambulatory confinement (HAMB; F_I_O_2_ = 0.142 ± 0.001; P_*I*_O_2_ = 90.4 ± 0.3 mmHg). Each 10-day experimental intervention required a commitment of 19 days from the participants, and comprised the following phases (see Figure 1): (1) the initial baseline testing phase comprising 5 days upon arrival to the facility [Basic Data Collection (BDC) was performed during this pre-intervention period]; (2) 10-day confinement phase (days 1–10) during which the participants were exposed to their designated condition (i.e., NBR, HBR, or HAMB); (3) a 4-day recovery phase allowing gradual reambulation of the participants following the BR interventions and enabling the researchers to perform the post-BDC period. All three experimental interventions (i.e., NBR, HBR, and HAMB) were conducted at the same time during 1 campaign such that 4 participants were enrolled in NBR, 4 in HBR and 4 in HAMB (see Figure 1). The campaigns were repeated until the participants completed all interventions which were separated by a minimum of a 1-month recovery/wash-out period. During each experimental intervention, polysomnography (PSG) was conducted on the first (night 1, N1) and tenth day (night 10, N10) of the confinement period. In addition, baseline sleep was assessed during the pre-BDC period of any one of the three interventions (i.e., NBR, HBR, or HAMB; see section “Sleep Monitoring” for more information). This baseline sleep recording was conducted on the third night that the participants had slept in their designated rooms. Thus, some degree of familiarisation to the sleeping arrangement had been achieved.

During the NBR and HBR interventions participants were confined to strict horizontal BR, a regularly employed ground-based experimental model to simulate microgravity-induced unloading. The details of the BR protocol, frequently employed by the group at the Planica facility, have been reported previously (McDonnell et al., 2020; Mekjavic et al., 2021). However, briefly, the participants were requested to conduct all daily activities in the horizontal position (i.e., reading, hygiene, watching television). One pillow was allowed for head support. Participants were allowed to change body positions from supine, prone, and lateral. No static or dynamic activity was permitted during the BR phase. 24-h closed-circuit television monitoring and permanent supervision of the medical staff ensured compliance with the experimental protocol and the safety and well-being of the participants.

During the HBR and HAMB interventions, the normobaric hypoxic ambient was established with a Vacuum Pressure Swing Adsorption system (b-Cat, Tiel, Netherlands) that delivered O_2_-depleted air to the designated rooms and hypoxic area. Air samples were obtained from each room at 15-min intervals, from which O_2_ and CO_2_ content was determined with O_2_ and CO_2_ analysers. These were calibrated with a reference gas prior to each 15-min measurement. The CO_2_ level within the confinement area did not exceed 0.5% throughout the interventions. As a safety precaution, during the hypoxic interventions (HBR and HAMB), each participant was issued a portable clip-on type O_2_ gas analyser with pre-set audible alarms (PGM-1100; RAE Systems, San Jose, CA, United States). These were always in close proximity at all times. During the HAMB intervention, the participants were encouraged to engage in their habitual routines and allowed to move freely in the common hypoxic area (±200 m^2^). They performed two 20-min low-intensity exercise sessions daily (morning and afternoon) to mimic their habitual physical activity routine. To avoid monotony, the activity modality (stepping, cycling, or dancing) was rotated daily. Before each intervention the participants performed a graded cycle ergometer test in normoxia (F_I_O_2_ = 0.209) and hypoxia (F_I_O_2_ = 0.142) to determine the appropriate physical activity intensity. The prescribed physical activity-intensity targeted to induce a heart rate (HR) corresponding to that attained at 50% of the hypoxia-specific maximum aerobic power. HR and capillary oxyhaemoglobin saturation (SpO_2_) were monitored continuously during the exercise sessions, using heart rate telemetry (iBody; Wahoo Fitness, Atlanta, GA, United States) and finger pulse oximetry (3100 WristOx; Nonin Medicals, Plymouth, MN, United States), respectively.

### Diet

The participants’ diet was reproduced so that in each intervention they received the same diet on each day of the respective interventions. Individualised energy requirements were calculated using the modified Harris-Benedict resting metabolic rate equation (Hasson et al., 2011) multiplied by a physical activity level factor of 1.2 for the NBR and HBR and 1.4 for the HAMB interventions. The targeted macronutrient composition was 55% carbohydrates, 30% fat, and 15% protein. The participants were supplemented with vitamin D3 (1,000 IU day^–1^) throughout all interventions and were encouraged to keep their daily fluid intake above 28.5 ml kg^–1^.

### Sleep Monitoring

To assess sleep, PSG was used. PSG was obtained using Nicolet One (Viasys, Healthcare, neurocare, Madison, WI, United States), and included electroencephalography, electrooculography, chin and tibial surface electromyography, electrocardiography, nasal pressure (nasal pressure cannula), respiratory movements (chest and abdominal belts), and capillary SpO_2_. Video surveillance during the night was implemented for safety, and to monitor the participants’ movements. A total of 7 PSG-examinations were planned for each participant: a baseline night, the 1st (NT1) and 10th (NT10) night of each intervention (NBR, HBR, and HAMB). PSG outcome measures that are included in the present study were performed by a single trained laboratory technician and are listed in Tables 1–5 (see also Tables 1–5 for a legend of all PSG-related abbreviations).

**TABLE 1:** Overview of sleep maintenance and efficiency outcomes in each level of both independent variables (i.e., activity and PO_2_).

	pNAMB	NBR	HAMB	HBR
	
	*M* ± *SD (n* = *8)*	*M* ± *SD (n* = *8)*	*M* ± *SD (n* = *10)*	*M* ± *SD (n* = *12)*
**Non-imputed data**				
TST (min)	353 ± 97	408 ± 34	399 ± 46	378 ± 57
SOL (min)	9 ± 7	23 ± 19	18 ± 15	33 ± 25
WASO (min)	35 ± 28	25 ± 23	55 ± 26	42 ± 24
EMA (min)	11 ± 11	30 ± 13	30 ± 18	42 ± 18
SE (%)	87 ± 6	84 ± 8	79 ± 8	76 ± 10

	**pNAMB**	**NBR**	**HAMB**	**HBR**
	
	***M* ± *SD (n* = *12)***	***M* ± *SD (n* = *12)***	***M* ± *SD (n* = *12)***	***M* ± *SD (n* = *12)***

**Imputed data**				
TST (min)	371 ± 84	395 ± 44	397 ± 42	378 ± 57
SOL (min)^	13 ± 9	32 ± 22	19 ± 14	33 ± 25
WASO (min)	54 ± 43	46 ± 50	56 ± 25	42 ± 24
EMA (min)+^	13 ± 9	31 ± 11	34 ± 18	42 ± 18
SE (%)+	87 ± 5	83 ± 6	78 ± 9	76 ± 10

*Mean (M) and standard deviation (SD) are depicted for both the data set with- and the data set without imputed data. Statistical analysis was performed on the imputed dataset only.*

*indicates a significant interaction effect between PO_2_ and activity, + indicates a significant main effect of PO_2_, ^ indicates a significant main effect of activity; PO_2_, partial pressure of oxygen; pNAMB, pseudo normobaric normoxic ambulatory; NBR, normobaric normoxic bed rest; HAMB, normobaric hypoxic ambulatory; HBR, normobaric hypoxic bed rest; TST, total sleep time; SOL, sleep onset latency; WASO, wake after sleep onset; EMA, early morning awakening (i.e., wakefulness after sleep offset); SE, sleep efficiency.

**TABLE 2:** Overview of sleep architecture outcomes in each level of both independent variables (i.e., activity and PO_2_).

	pNAMB	NBR	HAMB	HBR
	
	*M* ± *SD (n* = *8)*	*M* ± *SD (n* = *8)*	*M* ± *SD (n* = *10)*	*M* ± *SD (n* = *12)*
**Non-imputed data**				
N1 (% of TST)	11.3 ± 4.5	9.8 ± 5.3	19.5 ± 9.3	17.5 ± 11.4
N1 (min)	42 ± 21	39 ± 18	78 ± 42	64 ± 44
N2 (% of TST)	45.3 ± 8.6	48.9 ± 6.1	45.8 ± 11.3	48.3 ± 10.4
N2 (min)	160 ± 52	199 ± 26	184 ± 55	181 ± 49
N3 (% of TST)	31.3 ± 10.0	26.3 ± 6.1	18.8 ± 6.8	22.4 ± 5.7
N3 (min)	104 ± 23	108 ± 30	74 ± 25	86 ± 28
REM (% of TST)	12.2 ± 6.9	15.0 ± 7.1	16.0 ± 7.2	11.8 ± 5.5
REM (min)	48 ± 32	62 ± 30	63 ± 29	46 ± 24
REM latency (min)	142 ± 67	157 ± 58	166 ± 79	170 ± 73
N3 latency (min)	20 ± 11	21 ± 13	26 ± 13	31 ± 24
Alpha/delta sleep (# of intrusions)	25.1 ± 25.6	21.3 ± 11.2	34.6 ± 32.7	25.0 ± 22.6

	**pNAMB**	**NBR**	**HAMB**	**HBR**
	
	***M* ± *SD (n* = *12)***	***M* ± *SD (n* = *12)***	***M* ± *SD (n* = *12)***	***M* ± *SD (n* = *12)***

**Imputed data**				
N1 (% of TST)+	11.6 ± 3.6	11.4 ± 4.9	19.4 ± 8.5	17.5 ± 11.4
N1 (min)+	46 ± 18	41 ± 15	78 ± 38	64 ± 44
N2 (% of TST)^	43.2 ± 7.8	49.2 ± 5.3	44.5 ± 11.3	48.3 ± 10.4
N2 (min)	171 ± 48	202 ± 22	175 ± 54	181 ± 49
N3 (% of TST)*	29.4 ± 8.6	26.2 ± 4.9	19.2 ± 6.3	22.4 ± 5.7
N3 (min)+	101 ± 19	103 ± 26	73 ± 23	86 ± 28
REM (% of TST)	12.1 ± 7.3	14.8 ± 6.1	16.5 ± 6.9	11.8 ± 5.5
REM (min)	52 ± 34	58 ± 25	65 ± 27	46 ± 24
REM latency (min)	154 ± 63	171 ± 56	183 ± 84	170 ± 73
N3 latency (min)	21 ± 9	22 ± 10	26 ± 12	31 ± 24
Alpha/delta sleep (# of intrusions)	24.4 ± 20.8	21.4 ± 9.2	34.1 ± 29.7	25.0 ± 22.6

*Mean (M) and standard deviation (SD) are depicted for both the data set with- and the data set without imputed data. Statistical analysis was performed on the imputed dataset only.*

*Indicates a significant interaction effect between PO_2_ and activity, +indicates a significant main effect of PO_2_, ^ indicates a significant main effect of activity; PO_2_, partial pressure of oxygen; pNAMB, pseudo normobaric normoxic ambulatory; NBR, normobaric normoxic bed rest; HAMB, normobaric hypoxic ambulatory; HBR, normobaric hypoxic bed rest; N1%, percentage amount N1 of TST; N2%, percentage amount N2 of TST; N3%, percentage amount N3 of TST; REM%, percentage amount REM of TST; REM, rapid eye movement sleep; Alpha/delta sleep, number of intrusions of alpha activity during SWS.

**TABLE 3:** Overview of sleep fragmentation outcomes in each level of both independent variables (i.e., activity and PO_2_).

	pNAMB	NBR	HAMB	HBR
	
	*M* ± *SD (n* = *8)*	*M* ± *SD (n* = *8)*	*M* ± *SD (n* = *10)*	*M* ± *SD (n* = *12)*
**Non-imputed data**				
ArI (# of events/h of TST)	11.2 ± 3.8	10.5 ± 4.2	32.1 ± 16.6	31.4 ± 19.1
Arousals (# of events)	67 ± 31	72 ± 33	210 ± 110	190 ± 103
RERA (# of events)	38 ± 24	49 ± 33	165 ± 108	160 ± 92
MRA (# of events)	9 ± 6	11 ± 5	11 ± 7	9 ± 6
SAR (# of events)	21 ± 13	13 ± 7	34 ± 13	21 ± 13

	**pNAMB**	**NBR**	**HAMB**	**HBR**
	
	***M* ± *SD (n* = *12)***	***M* ± *SD (n* = *12)***	***M* ± *SD (n* = *12)***	***M* ± *SD (n* = *12)***

**Imputed data**				
ArI (# of events/h of TST) +	11.3 ± 3.3	10.7 ± 3.4	32.2 ± 15.0	31.4 ± 19.1
Arousals (# of events) +	66 ± 26	77 ± 28	203 ± 103	190 ± 103
RERA (# of events) +	39 ± 20	49 ± 27	163 ± 98	160 ± 92
MRA (# of events)	11 ± 6	12 ± 5	11 ± 7	9 ± 6
SAR (# of events) + ^	23 ± 11	14 ± 6	35 ± 12	21 ± 13

*Mean (M) and standard deviation (SD) are depicted for both the data set with- and the data set without imputed data. Statistical analysis was performed on the imputed dataset only.*

*Indicates a significant interaction effect between PO_2_ and activity, +indicates a significant main effect of PO_2_, ^indicates a significant main effect of activity; PO_2_, partial pressure of oxygen; pNAMB, pseudo normobaric normoxic ambulatory; NBR, normobaric normoxic bed rest; HAMB, normobaric hypoxic ambulatory; HBR, normobaric hypoxic bed rest; ArI, arousal index; Arousals, total number of arousals; RERA, respiratory-related arousals; MRA, movement-related arousals; SAR, spontaneous arousals.

**TABLE 4:** Overview of sleep-related respiration outcomes in each level of both independent variables (i.e., activity and PO_2_).

	pNAMB	NBR	HAMB	HBR
	
	*M* ± *SD (n* = *8)*	*M* ± *SD (n* = *8)*	*M* ± *SD (n* = *10)*	*M* ± *SD (n* = *12)*
**Non-imputed data**				
AHI (# of events/h of TST)	3.2 ± 2.2	3.0 ± 1.5	43.9 ± 47.8	30.5 ± 41.5
HI (# of events/h of TST)	1.8 ± 1.5	1.7 ± 0.9	14.3 ± 14.7	9.5 ± 7.3
OAI (# of events/h of TST)	0.7 ± 0.9	0.4 ± 0.3	1.9 ± 1.1	1.5 ± 1.2
CAI (# of events/h of TST)	0.7 ± 0.8	0.9 ± 0.6	27.5 ± 48.3	19.4 ± 38.9
MAI (# of events/h of TST)	0.00 ± 0.00	0.02 ± 0.04	0.21 ± 0.37	0.07 ± 0.10
RDI (# of events/h of TST)	8.8 ± 3.7	9.4 ± 4.7	54.5 ± 46.2	46.4 ± 40.2
ODI (# of events/h of TST)	2.1 ± 2.5	1.2 ± 1.1	44.4 ± 48.1	32.3 ± 42.2
MinSpO_2_%	90 ± 2	92 ± 1	75 ± 6	77 ± 10

	**pNAMB**	**NBR**	**HAMB**	**HBR**
	
	***M* ± *SD (n* = *12)***	***M* ± *SD (n* = *12)***	***M* ± *SD (n* = *12)***	***M* ± *SD (n* = *12)***

**Imputed data**				
AHI (# of events/h of TST) +	3.4 ± 1.8	3.2 ± 1.2	43.6 ± 43.2	30.5 ± 41.5
HI (# of events/h of TST) +	5.3 ± 7.6	4.1 ± 5.4	13.6 ± 13.5	9.5 ± 7.3
OAI (# of events/h of TST) +	1.2 ± 1.1	0.5 ± 0.3	1.9 ± 1.0	1.5 ± 1.2
CAI (# of events/h of TST) +	1.0 ± 0.8	1.1 ± 0.6	30.1 ± 44.5	19.4 ± 38.9
MAI (# of events/h of TST) + ^	0.00 ± 0.00	0.03 ± 0.03	0.25 ± 0.35	0.07 ± 0.10
RDI (# of events/h of TST) +	9.1 ± 3.2	9.9 ± 3.8	55.8 ± 42.2	46.4 ± 40.2
ODI (# of events/h of TST) +	3.1 ± 2.5	1.3 ± 0.9	44.8 ± 43.5	32.3 ± 42.2
MinSpO_2_% +	90 ± 2	92 ± 1	75 ± 5	77 ± 10

*Mean (M) and standard deviation (SD) are depicted for both the data set with- and the data set without imputed data. Statistical analysis was performed on the imputed dataset only.*

*Indicates a significant interaction effect between PO_2_ and activity, +indicates a significant main effect of PO_2_, ^ indicates a significant main effect of activity; PO_2_, partial pressure of oxygen; pNAMB, pseudo normobaric normoxic ambulatory; NBR, normobaric normoxic bed rest; HAMB, normobaric hypoxic ambulatory; HBR, normobaric hypoxic bed rest; AHI, apnea-hypopnea index; HI, hypopnea index; OAI, obstructive apnea index; CAI, central apnea index; MAI, mixed apnea index; RDI, respiratory disturbance index (RDI = AHI + [RERA/h]); ODI, oxygen desaturation index (ODI = number > 3% drops in SpO_2_/h); MinSpO_2_%, minimal blood oxygen saturation.

**TABLE 5:** Overview of periodic leg movement outcomes in each level of both independent variables (i.e., activity and PO_2_).

	pNAMB	NBR	HAMB	HBR
	
	*M* ± *SD (n* = *8)*	*M* ± *SD (n* = *8)*	*M* ± *SD (n* = *10)*	*M* ± *SD (n* = *12)*
**Non-imputed data**				
PLMSI (# of events/h of TST)	0.08 ± 0.21	0.29 ± 0.70	0.46 ± 0.63	2.36 ± 2.85

	**pNAMB**	**NBR**	**HAMB**	**HBR**
	
	***M* ± *SD (n* = *12)***	***M* ± *SD (n* = *12)***	***M* ± *SD (n* = *12)***	***M* ± *SD (n* = *12)***

**Imputed data**				
PLMSI (# of events/h of TST)	0.16 ± 0.22	0.42 ± 0.60	0.52 ± 0.59	2.36 ± 2.85

*Mean (M) and standard deviation (SD) are depicted for both the data set with- and the data set without imputed data. Statistical analysis was performed on the imputed dataset only.*

*Indicates a significant interaction effect between PO_2_ and activity, +indicates a significant main effect of PO_2_, ^ indicates a significant main effect of activity; PO_2_, partial pressure of oxygen; pNAMB, pseudo normobaric normoxic ambulatory; NBR, normobaric normoxic bed rest; HAMB, normobaric hypoxic ambulatory; HBR, normobaric hypoxic bed rest; PLMSI, periodic leg movement during sleep index.

The baseline PSG-recording was only performed after the participants spent for two nights in the facility during the initial BDC testing phase. The baseline PSG-recording was performed once for each participant such that for the first four participants this took place in the BDC phase before their first condition, for the fifth to the eighth participant, before their second condition and finally, for the ninth to the twelfth participant, before their third and last condition. All conditions were separated by a 1-month recovery period. No PSG-habituation night was included, however, due to the randomisation of both the conditions and the baseline night, a plausible first night-effect should be filtered out (i.e., one does always need to adapt to sleeping with PSG-equipment, certainly during the first night).

Sleep recordings were visually analysed, and scored for sleep and respiration following the European Sleep Research Society guidelines and the American Association for Sleep Medicine guidelines (Berry et al., 2012). Hypopneas were defined as a drop ≥30% (i.e., compared to pre-event baseline) in air flow for ≥10 s that was associated with ≥4% desaturation, and apneas were defined as a reduction of ≥90% for ≥10 s. Participants maintained standard sleep-wake cycles throughout the study, with “lights off” at 2300 hours and “lights on” at 0700 hours. They were not allowed to nap during the day, and were not permitted to consume alcohol, caffeine, drugs, or other stimulants. Participants were excluded from the sleep study if, after baseline recordings, it was discovered that they had significant periodic limb movements (PLM index > 5/h), obstructive sleep apnea [apnea-hypopnea index (AHI) > 5], or other sleep abnormalities. No participants exhibited such behaviours and, as such, no participants were excluded based on one of these reasons.

### Statistics

#### Statistics Focusing on a Possible Interaction Between Hypoxia and BR

The first statistical objective of this study was to evaluate the interaction between partial pressure of oxygen (PO_2_) and activity on sleep characteristics in women. Therefore, the baseline PSG-recording that was performed once for each participant throughout their participation in this study (see section “Sleep Monitoring” for additional information) was included as a pseudo fourth condition (i.e., pseudo normobaric normoxic ambulatory, pNAMB) in the present study. Unlike the other three conditions (i.e., NBR, HBR, and HAMB), pNAMB did not require a 19-day commitment from the participants. The baseline PSG-recording was interpreted as the pNAMB-condition and therefore there is only data for pseudo night 1 in pNAMB (i.e., the baseline PSG-recording), while data for pseudo night 10 is missing in the pNAMB condition. To reduce the relative amount of missing data and solely focus on the effects of PO_2_ and activity, the factor “time” (1st vs 10th night) was not accounted for in this main analysis. To filter out the time factor in our dataset, a mean of the first and tenth night was taken for each individual in each of the four conditions and this resulted in 48 data values and two independent variables that remained (i.e., PO_2_ and activity). For the pNAMB-condition (i.e., the pseudo condition where only one PSG-night was performed), and for the individuals that were not able to complete two PSG-nights in any of the other conditions (i.e., NBR, HAMB and HBR), the last measurement was carried forward as the mean. Following this procedure, 10 out of 48 (i.e., 20.8%; missing at random) data values were still missing for each outcome parameter. Prior to this averaging-procedure, 34 out of 96 data values were missing (i.e., 35%; missing at random) for each outcome parameter. Data were missing due to participant drop-out or breakdown of PSG-device. Data that were still missing were estimated by multiple imputations (Newman, 2014; Hair et al., 2018). In phase 1 (imputation phase), the available data per outcome variable were used to impute 40 data sets (fully conditional specification imputation method) (Newman, 2014). Subsequently, in phase 2 (pooling phase), the parameter estimates were averaged across the 40 imputed data sets to get the final parameter estimates. The main analysis was carried out with this data set. Based on the guidelines of Sterne et al. (2009) a Supplementary Table 1 was created to compare the mean and standard deviation of all outcome parameters between participants with complete (*n* = 7) and incomplete data (*n* = 5). Supplementary Table 1 demonstrates that no important differences between participants with complete and incomplete data are present and that the mean of both groups is comparable and falls within the 95%-confidence interval.

The main analysis included a Shapiro-Wilk test and visual interpretation of histograms to test the normality of the data. Sphericity was verified by Mauchly’s test. When the assumption of sphericity was not met, the significance of F-ratios was adjusted with the Greenhouse-Geisser procedure. If data were not normally distributed (i.e., TST, WASO, N3 latency, alpha/delta sleep, spontaneous micro-arousals, AHI, HI, CAI, MAI, MinSpO_2_, ODI, and PLMSI; see Tables 1–5 for a legend of all abbreviations), a square root transformation was employed for positively skewed data to attain normally distributed data (this was successful for: WASO, AHI, HI, CAI, ODI, N3 latency, and alpha/delta sleep) and for negatively skewed data to attain normally distributed data a square root transformation was performed after subtracting the data from a constant factor that was one unit higher than the maximum observed value (this was successful for: TST). If the square root transformation did not result in normally distributed data (this was the case for: spontaneous micro-arousals, MAI, PLMSI and MinSPO_2_), non-parametric Wilcoxon tests were used to observe the effect of PO_2_ and activity. All other parameters (see Tables 1–5) were normally distributed. The effect of PO_2_ (normoxia vs hypoxia) and activity (ambulatory vs bed rest) on all normally distributed parameters was assessed by a two-way repeated-measures ANOVA. If a significant interaction effect was observed, subsequent paired-samples *t*-tests were performed to elucidate the main effect of PO_2_ and activity. If no significant interaction effect was observed (i.e., no *post hoc* paired-samples *t*-tests were performed), then the main effects of PO_2_ and activity were further interpreted through pairwise comparisons with a Bonferroni correction. Significance was set at <0.05 for all analyses, which were conducted using the Statistical Package for the Social Sciences, version 27 (SPSS Inc., Chicago, IL, United States).

A supplemental analysis was performed to evaluate whether timing of the baseline PSG-recordings had an effect on the sleep characteristics. Namely, four participants conducted the PSG measurements prior to the first intervention, whereas the remaining eight conducted their PSG measurements prior to either their second or third intervention. To test for the effect of timing of the PSG measurements, a non-parametric Mann–Whitney U Test, with the timing of the baseline PSG-recording (i.e., four participants vs eight participants) taken as the between subject factor, was performed to indicate whether the timing of the baseline PSG-recording impacted the polysomnographic data.

#### Statistics Focusing on the Pre-defined Hypotheses

The second statistical objective of this study was to assess possible sex-specific differences in the effects of PO_2_ and activity on sleep characteristics by formulating pre-defined hypotheses based on the findings reported by Rojc et al. (2014) (i.e., The LunHab project, an identical version of the current project except with men). In order to attain this second statistical objective, the factor ‘time’ (1st vs 10th night) was this time accounted for in the analysis.

The pre-defined hypotheses were: (1) Hypoxia reduces the amount of N3 sleep and increases N2 sleep, (2) Sleep stage values return to baseline levels after sustained hypoxic exposure (i.e., 10 days) when active (i.e., ambulatory), but not when inactive (i.e., BR), (3) BR increases WASO compared to baseline, and (4) WASO returns to baseline after prolonged exposure to BR.

Shapiro–Wilk tests revealed that all included data was normally distributed. Therefore, to statistically assess these pre-defined hypotheses, paired-samples *t*-tests were performed, as described below.

Hypothesis (H) 1: Two paired-samples t-tests were performed for both N3 sleep and N2 sleep, the first *t*-test included the baseline and the night 1 sleep recording of HAMB, while the second *t*-test included the same data sets but from HBR (see Table 6).

**TABLE 6:** Overview of all polysomnography-related data required to evaluate the pre-defined hypotheses that were based on the reported results in the study of Rojc et al. (2014).

Non-imputed data							

	pNAMB	NBR		HAMB		HBR	

	(*n* = *8)*	*Night 1* (*n* = *8)*	*Night 10* (*n* = *7)*	*Night 1* (*n* = *10)*	*Night 10* (*n* = *8)*	*Night 1* (*n* = *11)*	*Night 10* (*n* = *10)*
	*M* ± *SD*	*M* ± *SD*	*M* ± *SD*	*M* ± *SD*	*M* ± *SD*	*M* ± *SD*	*M* ± *SD*
*Hypothesis 1; the acute effect of hypoxia on sleep architecture*							
N2 (min)	160 ± 52			191 ± 52		181 ± 58	
N3 (min)	104 ± 23			73 ± 26		78 ± 37	
*Hypothesis 2; the effect of sustained hypoxic exposure on sleep architecture*							
N1 (min)	42 ± 21				79 ± 58		63 ± 38
N3 (min)	l104 ± 23				77 ± 28		88 ± 25
*Hypothesis 3; the effect of BR on the number of awakenings*							
Arousals (# of events) ^°	67 ± 31	76 ± 48		205 ± 128		181 ± 119	
*Hypothesis 4; the effect of sustained BR on the number of awakenings*							
Arousals (# of events)°	67 ± 31		71 ± 38				221 ± 119

**Imputed data**							

	**pNAMB**	**NBR**		**HAMB**		**HBR**	

	**(*n* = *12)***	***Night 1*** **(*n* = *12)***	***Night 10*** **(*n* = *12)***	***Night 1*** **(*n* = *12)***	***Night 10*** **(*n* = *12)***	***Night 1*** **(*n* = *12)***	***Night 10*** **(*n* = *12)***
	***M* ± *SD***	***M* ± *SD***	***M* ± *SD***	***M* ± *SD***	***M* ± *SD***	***M* ± *SD***	***M* ± *SD***

*Hypothesis 1; the acute effect of hypoxia on sleep architecture*							
N2 (min)	163 ± 42			189 ± 47		181 ± 55	
N3 (min) ^°	97 ± 22			73 ± 23		79 ± 36	
*Hypothesis 2; the effect of sustained hypoxic exposure on sleep architecture*							
N1 (min) ^	47 ± 20				88 ± 50		66 ± 35
N3 (min) ^	97 ± 22				78 ± 24		91 ± 23
*Hypothesis 3; the effect of BR on the number of awakenings*							
Arousals (# of events) ^°	73 ± 28	92 ± 45		214 ± 118		181 ± 113	
*Hypothesis 4; the effect of sustained BR on the number of awakenings*							
Arousals (# of events)°	73 ± 28		71 ± 28				208 ± 113

*Mean (M) and standard deviation (SD) are depicted for both the data set with- and the data set without imputed data.*

*+Indicates a significant difference between pNAMB and NBR, ^indicates a significant difference between pNAMB and HAMB, °indicates a significant difference between pNAMB and HBR; pNAMB, pseudo normobaric normoxic ambulatory; NBR, normobaric normoxic bed rest; HAMB, normobaric hypoxic ambulatory; HBR, normobaric hypoxic bed rest; Arousals, total number of arousals.*

H 2: Two paired-samples *t*-tests were performed for both N3 and N1 sleep, data included in these paired-samples *t*-tests was: baseline and night 10 sleep recording for both HAMB and HBR (see Table 6).
H 3: A paired-samples *t*-test comparison was made between the number of awakenings during baseline and night 1 in each of the interventions (NBR, HBR, and HAMB, see Table 6).
H 4: A paired-samples *t*-test comparison between the number of awakenings during baseline and that of night 10 of both NBR and HBR.

Similarly to the above-mentioned analysis that focussed on a possible interaction between PO_2_ and activity, missing data values had to be handled here as well. To assess whether the imputation of missing data significantly impacted the results, the paired-samples *t*-test that are outlined in the current paragraph were performed once with the imputed data set and once without.

## Results

### Possible Interaction Between PO_2_ and Activity

See Tables 1–5 for all sleep-related data.

#### Sleep Maintenance and Efficiency

Regarding sleep maintenance, participants showed significantly higher early morning arousal duration due to both hypoxia [main effect of PO_2_; *F*(1,11) = 13.1, *p* = 0.004, η_p_^2^ = 0.544] and BR [main effect of activity; *F*(1,11) = 8.5, *p* = 0.014, η_p_^2^ = 0.436, see Table 1]. Moreover, independently from PO_2_, BR also increased SOL [main effect of activity; *F*(1,11) = 16.8, *p* = 0.002, η_p_^2^ = 0.605]. In general, the average sleep efficiency in normoxic conditions (i.e., pNAMB and NBR) reached normal levels (estimated mean of 85%; Walsleben et al., 2004), but dropped significantly in hypoxia [estimated mean of 77%; main effect of PO_2_; *F*(1,11) = 7.6, *p* = 0.019].

#### Sleep Architecture

**FIGURE 2:**
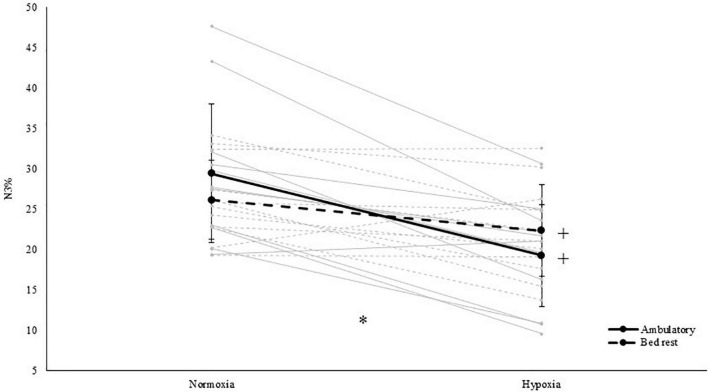
Relative percentage of slow wave sleep (i.e., N3% of total sleep time) in both normoxia and hypoxia and when being active (i.e., ambulatory) and inactive (i.e., BR). *Represents a significant interaction between activity and PO_2_. +represents a significant difference between normoxia and hypoxia in that specific level of activity. Solid grey lines represent the individual response to hypoxia in an ambulatory condition, dashed grey lines represent the individual response to hypoxia in a BR condition.

With respect to sleep architecture, our results showed that light sleep (i.e., N1) significantly increased both in duration [main effect of PO_2_; *F*(1,11) = 5.6, *p* = 0.038, η_p_^2^ = 0.336; see Table 2] and in proportion of TST [main effect of PO_2_; *F*(1,11) = 6.2, *p* = 0.030, η_p_^2^ = 0.360; see Table 2] during hypoxic exposure. Whereas BR increased the N2 sleep percentage of TST [main effect of activity; *F*(1,11) = 6.0, *p* = 0.033, η_p_^2^ = 0.351]. In addition, hypoxia significantly decreased N3 sleep duration by 22 min [main effect of PO_2_; *F*(1,11) = 14.4, *p* = 0.003, η_p_^2^ = 0.567; see Table 2]. In terms of the percentage N3 (i.e., slow wave sleep) of TST, an interaction effect between PO_2_ and activity was observed [*F*(1,11) = 14.2, *p* = 0.003, η_p_^2^ = 0.563]. Follow-up paired-samples *t*-tests revealed that hypoxia decreased N3% both when HAMB was compared to pNAMB (*p* < 0.001, *t* = 6.0) and when HBR was compared to NBR (*p* = 0.021, *t* = 2.7). While BR appeared to decrease N3% in normoxia (pNAMB vs NBR; *p* = 0.088, *t* = 1.9) and increase N3% in hypoxia (HAMB vs HBR; *p* = 0.060, *t* = –2.1; see Figure 2), no significant effects were observed. No interaction and/or main effects were observed for REM sleep, REM latency, N3 latency or alpha/delta sleep.

#### Sleep Fragmentation

The current analyses revealed that micro-arousals significantly increased both in absolute terms [main effect of PO_2_; *F*(1,11) = 24.6, *p* < 0.001, η_p_^2^ = 0.691; see Table 3] and in relation to TST [main effect of PO_2_; *F*(1,11) = 25.7, *p* < 0.001, η_p_^2^ = 0.700; see Table 3] in hypoxia. More specifically, total sleep fragmentation increased during hypoxic exposure (i.e., both in HBR and HAMB), mainly due to an increase in spontaneous (pNAMB vs HAMB: *p* = 0.084; NBR vs HBR: *p* = 0.041) and respiratory-related arousals [main effect of PO_2_; *F*(1,11) = 26.4, *p* < 0.001, η_p_^2^ = 0.706; see Table 3]. In terms of activity, BR was found to decrease (i.e., positively impact) spontaneous arousals (pNAMB vs NBR: *p* = 0.003; HAMB vs HBR: *p* = 0.014; see Table 3).

#### Sleep-Related Respiration

**FIGURE 3:**
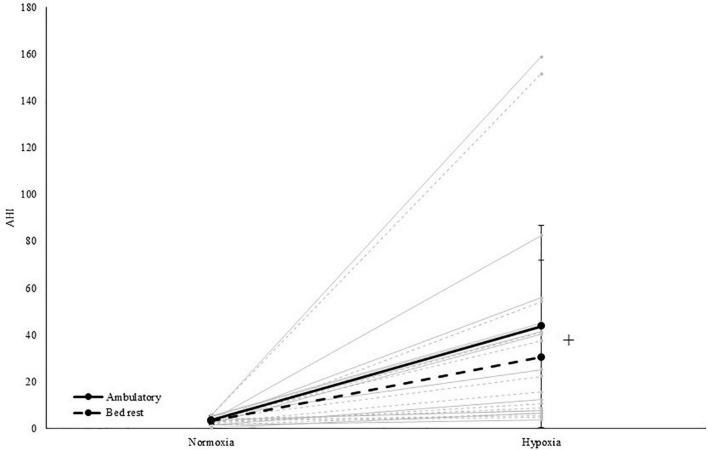
Apnea-hypopnea index in both normoxia and hypoxia and when being active (i.e., ambulatory) and inactive (i.e., BR). +Represents a significant main effect of PO_2_. Solid grey lines represent the individual response to hypoxia in an ambulatory condition, dashed grey lines represent the individual response to hypoxia in a BR condition.

The AHI increased in hypoxia (37 events/h) compared to normoxia [<5 events/h; main effect of PO_2_; *F*(1,11) = 25.1, *p* < 0.001, η_p_^2^ = 0.695; see Table 4 and Figure 3]. More specifically, obstructive events [main effect of PO_2_; OAI; *F*(1,11) = 25.3, *p* < 0.001, η_p_^2^ = 0.697], central events [main effect of PO_2_; CAI; *F*(1,11) = 10.4, *p* = 0.008, η_p_^2^ = 0.486], hypopneas [main effect of PO_2_; HI; *F*(1,11) = 118.2, *p* < 0.001, η_p_^2^ = 0.915] and RDI [main effect of PO_2_; *F*(1,11) = 15.0, *p* = 0.003, η_p_^2^ = 0.576] significantly increased during hypoxic exposure. The MAI-data was not normally distributed and Wilcoxon tests indicated that MAI increased due to hypoxia, specifically in ambulatory conditions (pNAMB vs HAMB; *p* = 0.017). In addition, BR increased MAI during normoxic exposure (pNAMB vs NBR; *p* = 0.028). In hypoxia, oxygen saturation expectedly decreased - on average below 80% SpO_2_ (pNAMB vs HAMB: *p* = 0.002, NBR vs HBR: *p* = 0.002; see Table 4)— compared to in normoxia. Moreover, the ODI was also significantly higher [main effect of PO_2_; ODI; *F*(1,11) = 27.7, *p* < 0.001, η_p_^2^ = 0.716] in hypoxia compared to normoxia.

#### Periodic Leg Movements

Periodic leg movements were not impacted by PO_2_ or activity, and no significant interaction effect was found. Mean indices remained below clinical threshold values (Berry et al., 2015).

#### Effect of Timing of the Baseline PSG-Recording

The analysis of the effect of the timing of the baseline PSG-recordings revealed that the pNAMB-data of the four participants that performed the baseline PSG-recording in the BDC phase before their first condition, significantly differed from the pNAMB-data of the other eight participants, who performed the baseline PSG-recording in the BDC phase either before their second or third condition. A significant difference was found for sleep efficiency (*p* = 0.027), N1% (*p* = 0.027), and OAI (*p* = 0.040; see Supplementary Table 2), while all other polysomnographic outcomes did not significantly differ (see Supplementary Table 2).

### The Pre-defined Hypotheses

#### Hypothesis 1: The Acute Effect of Hypoxia on Sleep Architecture

In the non-imputed data set, despite a noticeable drop in N3 sleep duration in HAMB and HBR compared to pNAMB (see Table 6), paired-samples *t*-tests did not reveal any difference in N3 sleep duration between pNAMB (i.e., the baseline recording) and night 1 in HAMB or HBR. In the imputed data set, a similar noticeable drop in N3 duration during night 1 in HAMB [*p* = 0.009, *t*(11) = 3.2, cohen’s d (d) = 0.911] and HBR [*p* = 0.036, *t*(11) = 2.4, d = 0.688] compared to pNAMB was found to be significant (see Table 6). No effects were observed for N2 duration.

#### Hypothesis 2: The Effect of Sustained Hypoxic Exposure on Sleep Architecture

To assess the effect of sustained hypoxic exposure on sleep architecture, paired-samples *t*-tests were performed between the pNAMB and the night 10-recording in HAMB and HBR. There were no significant differences found within the non-imputed data set, while in the imputed data set, N1 duration significantly increased [*p* = 0.036, *t*(11) = –2.4, d = –0.689], and N3 duration [*p* = 0.026, t(11) = 2.6, d = 0.744] decreased in HAMB compared to pNAMB (see Table 6).

#### Hypothesis 3: The Effect of BR on the Number of Awakenings

Within both the imputed and non-imputed data sets, WASO significantly increased during night 1 in HAMB [non-imputed: *p* = 0.041, t(6) = –2.6, d = –0.983; imputed: *p* = 0.002, t(11) = –4, d = –1.155] and HBR [non-imputed: *p* = 0.027, t(7) = –2.8, d = –0.981; imputed: *p* = 0.004, t(11) = –3.6, d = –1.034] compared to in pNAMB. No difference was observed between pNAMB and NBR (see Table 6).

#### Hypothesis 4: The Effect of Sustained BR on the Number of Awakenings

Sustained BR resulted in a significant increase in WASO during night 10 in HBR [pNAMB vs HBR; non-imputed: *p* = 0.009, t(5) = –4.1, d = –1.668; imputed: *p* = 0.001, t(11) = –4.6, d = –1.333] but not in NBR (see Table 6).

## Discussion

The present repeated measures cross-over study with women assessed the effect of reduced PO_2_ (i.e., normobaric hypoxia) and inactivity (i.e., BR) on sleep, and demonstrated that hypoxia negatively impacted multiple aspects of sleep (i.e., sleep maintenance and efficiency, sleep architecture, sleep fragmentation and sleep-related respiration; see Tables 1–4). Additionally, BR specifically impaired sleep maintenance and efficiency (see Table 1). There was a significant interaction between PO_2_ and activity, which revealed that hypoxia decreased N3% independently from whether participants were active (i.e., ambulatory) or inactive (i.e., BR). However, this hypoxia-related decrease was attenuated when they were inactive (NBR vs HBR; -3.8%) compared to when they were active (pNAMB vs HAMB; -10.2%). The current results were compared to those reported by our group from a similar study conducted with men (Rojc et al., 2014). Sex-specific differences in the acclimatisation to prolonged exposure to hypoxia and BR exist. Women did not acclimate to either hypoxia or BR in terms of sleep architecture or WASO, while the men did (Rojc et al., 2014).

### The Effect of Hypoxia and BR on Sleep

The normobaric hypoxic conditions (HBR and HAMB equivalent to an altitude of 4,000 m) of the present study created similar significant effects on sleep architecture (especially on sleep maintenance, efficiency, and deep sleep to light sleep exchange) to those previously reported (Bloch et al., 1985; Anholm et al., 1992). The effect of acute hypobaric and normobaric hypoxia on sleep architecture has been generally observed as a significant decrease in SWS (i.e., N3 sleep) and an increase in the lighter NREM sleep stages, N1 and N2 (Bloch et al., 1985). This is in line with our findings where both the significant decrease in SWS and increase in N1 were observed during hypoxic exposure, independently from whether they were active (i.e., ambulatory) or inactive (i.e., BR). Anholm et al. (1992) found that hypoxia-related respiratory disturbances could underlie the often-observed impairments in sleep when residing in a hypoxic environment. Furthermore, a respiratory-related increase in sleep fragmentation has also been observed in personnel at the Concordia Antarctic research station, situated at an altitude of approximately 3,200 m (Fernandez Tellez et al., 2014). Due to the lower pressure at the poles, the P_*I*_O_2_ in the Concordia station is similar to the one simulated in the present study. The role of respiratory disturbances in the hypoxia-induced deteriorations in sleep parameters is further confirmed in the present study. Obstructive and central events, hypopneas and the RDI significantly increased during hypoxic exposure. Despite the present study indicating an important role of respiratory disturbances in the hypoxia-induced deteriorations in sleep parameters, other factors will undoubtedly also underly the negative impact of hypoxia on sleep (e.g., blood oxygen saturation; Nussbaumer-Ochsner et al., 2012).

During BR the most affected sleep parameters were sleep maintenance, efficiency, and sleep architecture. The current finding of an increase in N2% (negative effect on sleep architecture) is in line with previous findings by Rojc et al. (2014) and Morrison et al. (2017) in men. Similarly to the present female study, Morrison et al. (2017) observed in their study that BR increased the percentage amount of light sleep in men. However, in the present study, BR also led to an increase in SOL and EMA’s, findings that were not observed by Morrison et al. (2017). Rojc et al. (2014), however, did find an increase in the number of awakenings due to BR. In addition, Monk et al. (1997), who conducted a study with only men, observed that BR increased SOL. However, SOL was recorded through a self-reported sleep diary and not via an objective measurement (i.e., PSG) like in the present study. Taken together, it appears that the present study provides evidence that women experience similar negative effects of BR on sleep as those of their male counterparts.

The reinforcing interaction between PO_2_ and activity that was previously suggested by the data reported in the study of Morrison et al. (2017) was not observed in the present study. In contrast, the current study found that BR attenuated the negative effect of hypoxia on sleep macrostructure. This attenuating interaction corroborates a conclusion that was previously put forward by our group (Tellez et al., 2016), i.e., that performing physical exercise can exacerbate the number of nocturnal apneas and hypopneas when residing at altitude. To explain the negative effect of exercise on sleep at altitude, Tellez et al. (2016) argued that exercise-evoked sympathetic activation increases chemoreceptor sensitivity and subsequently also further increases the occurrence of periodic breathing at altitude. In the study of Hermand et al. (1985) a solid indication of this suggested interaction between hypoxia and exercise in terms of breathing pattern was presented. They reported that, at the same exercising ventilation level, oscillations of ventilation [i.e., oscillations in the length (in seconds) and peak power (in L^2^ min^–2^) of the oscillations that characterise periodic breathing] were much greater in hypoxia than in normoxia (Hermand et al., 1985). These results indicate that periodic breathing was aggravated when performing exercise at altitude. Moreover, the mechanisms thought to underly the exercise-evoked periodic breathing at altitude (i.e., sympathetic activation) can persist for up to 6 days after an exercise session is completed (Eyzaguirre and Lewin, 1961). Meaning that during the nights following on the exercise session the negative interaction effect of physical exercise and hypoxia might also persist. In the present study, participants performed two 20-min low-intensity daily physical activity sessions (morning and afternoon) in HAMB, to mimic their habitual activity. Based on the above-mentioned reasoning we suggest that these daily physical activity sessions might underly the interaction that was observed between PO_2_ and activity, i.e., during hypoxic exposure, the daily physical activity sessions may further increase the chronic sympathoexcitation that is linked with high altitude exposure (Mazzeo and Reeves, 2003; Lundby et al., 2018), subsequently triggering periodic breathing and eventually impairing sleep macrostructure more in HAMB than in HBR.

### Women and Sleep

With very few exceptions (Szymczak et al., 2009; Johnson et al., 2010; Frost et al., 2021), sleep studies conducted at altitude have included primarily men (Pattyn et al., 1985; Bolmont et al., 2000; Stadelmann et al., 2013; Fernandez Tellez et al., 2014; Rojc et al., 2014; Morrison et al., 2017; Bian et al., 2019; Mairesse et al., 2019). This is remarkable, as it is clear that sex does lead to significant differences in the outcomes of psychophysiological research (Mark et al., 2014). Specifically in terms of sleep, sex-specific differences have been demonstrated in sleep duration and WASO (Jonasdottir et al., 2021). Moreover, women have been found to exhibit phase advanced core body temperature and melatonin rhythms, as well as a shorter intrinsic circadian period compared with men (Cain et al., 2011; Duffy et al., 2011). Of course, sex-specific differences do not only exist in sleep, they may also exist in terms of the effect of BR and hypoxia on sleep (Mark et al., 2014). Unlike the substantial normalisation of sleep architecture following 10 days HAMB or HBR seen in LunHab (Rojc et al., 2014), the present study observed that this normalisation was absent in women. Compared to baseline, a significant reduction of N3 sleep duration and an increase of light sleep-duration (i.e., N1 and/or N2) was observed both during night 1 and night 10 in HAMB. The return of WASO to baseline after acclimating during a 10 day BR observed in LunHab (Rojc et al., 2014) was not found in the present female cohort. Thus, indicating that sex-specific differences in the effect of hypoxia and BR on sleep do exist. While, there are indeed some sex-specific differences in the acclimatisation process to BR and hypoxia, the acute effects observed in the present study were generally similar to the identical male-study (LunHab) (Rojc et al., 2014). Both men and women exhibited a significant hypoxia-related decrease in SWS (i.e., N3%) and an increase in light sleep. In addition, BR was found to decrease sleep maintenance and efficiency in both men and women. The impact of imputing missing data on the above-mentioned results was evaluated by conducting the statistical analysis once with the non-imputed data set and once with the imputed data set. This revealed that the statistical results from both kinds of analyses were largely comparable and indicated that the imputations were non-decisive in the statistical results.

Besides specific differences in sleep between sexes, sleep has also been found to differ within women themselves, based on the menstrual phase (Baker and Lee, 2018). Indeed, previous research has observed overall subjective sleep disturbances during the premenstrual and menstruation phases (Baker and Lee, 2018). In addition, it has also been shown that an altered menstrual cycle disrupts sleep (Baker and Lee, 2018). Furthermore, the usage of oral contraceptives has been known to trigger an increase in light sleep N2, a decrease in SWS and significant core body temperature changes, seeing a significant increase throughout the nocturnal sleep period (Baker and Lee, 2018). In the present study, we did not regulate the use of oral contraceptives, nor did we conduct the interventions so that they would coincide with a particular phase of the menstrual cycle. As a result, we cannot comment on whether the menstrual cycle significantly impacted the effect of hypoxia and BR on sleep. However, based on the results reported by Shechter et al. (2011) it seems that the menstrual cycle is not a main moderator of the effect BR on sleep. Shechter et al. (2011) examined sleep onset and propensity during 36 cycles of 60-min wake periods and 60-min nap opportunities during a 5-day constant BR protocol, and reported that different phases of the menstrual cycle showed little to no effect on sleep behaviour. Shechter et al. (2011) found that women with regular menstruation cycles, had similar sleep propensity throughout all phases of the menstrual cycle. Sleep disturbances during premenstrual and menstrual phases have been reported for women with painful menstrual cramps (Baker and Lee, 2018), but no such cramps were reported in our group of participants.

### Future Research

The underlying mechanism of the negative impact of BR and certainly hypoxia on sleep appears to be respiratory-related. Respiratory-related issues have been suggested to arise due to peripheral hypoxic chemosensitivity (Tellez et al., 2016). In the study of Nespoulet et al. (2012) it was concluded that peripheral hypoxic chemosensitivity appears to be a major causative factor for altitude intolerance (i.e., the lower the peripheral hypoxic chemosensitivity, the lower one’s night SpO_2_). The results of Nespoulet et al. (2012) indicated that acute mountain sickness-susceptible patients’ night SpO_2_ concentrations were ∼5% lower than their non-susceptible counterparts while exposed to a simulated altitude of ∼3,000 m, yet these patients experienced a significantly lower AHI (i.e., higher breathing stability; 18 vs. 33 events per hour; *p* = 0.038). These results indicate that a focus on interindividual differences in the effect of hypoxia and BR on sleep would most certainly increase our insight in the underlying mechanisms of the hypoxia- and BR-related deteriorations in sleep. The stability of interindividual differences was not evaluated in our study due to the large intraindividual variability (i.e., the variability between the repeated measures within each individual).

Another interesting factor that could create further insight in the impact of hypoxia and bed rest on sleep is circadian typology (such as melatonin and cortisol secretion measures). In a recent study by Calderon-Jofre et al. (2021), the effect of chronic intermittent hypobaric hypoxia on sleep quality and morning melatonin serum levels in Chilean miners was assessed and an altitude-associated increase in melatonin serum levels was observed. Moreover, an inverse correlation between melatonin concentration and nocturnal oxygen saturation was found (Calderon-Jofre et al., 2021). These findings indicate that altitude-associated impairments in sleep could be related to changes in melatonin levels. Circadian phenotyping and entrainment could add valuable information on individual differences in tolerance to hypoxia and BR when looking at sleep, which as we know has a crucial impact on daytime functioning.

Lastly, to rigorously assess the possible habituation effects, longer exposure-duration studies are necessary [e.g., the study of Morrison et al. (2017)]. Within these longer exposure-duration studies it is required to also include women to provide the opportunity to evaluate sex-specific differences as well as menstrual-cycle related differences within women. In addition, longer exposure-duration studies would also provide the opportunity to evaluate whether or not high altitude-habituation counteracts the negative interaction between activity and PO_2_ that was observed in the present study. Tellez et al. (2016) demonstrated that, regardless of habituation, physical activity *per se* increases the incidence of periodic breathing in both hypobaric and normobaric hypoxic environments. However, the evidence of a possible interaction effect between activity and PO_2_ remains equivocal (Berssenbrugge et al., 1983; Mazzeo and Reeves, 2003; Bloch et al., 2010).

### Limitations

The lack of an *a priori* sample size calculation is the first limitation of our study. The current study was conducted with a convenience sampling of 12 participations. No previous studies investigating the possible interaction effect of hypoxia and BR had been performed at the time this study was designed and therefore no valid effect size was present in the published literature. Therefore, similar to the male study (Rojc et al., 2014), it was decided to go for a convenience sampling of 12 participants.

Data were missing to some extent (i.e., 20.8%) from the data set that was analysed and interpreted in the current study. An array of approaches to handle the issue of missing data have been described, with multiple imputation being one of them (Carpenter and Kenward, 2008; Sterne et al., 2009). Multiple imputation is an approach in which different imputed data sets are created and eventually combined to estimate the missing values (see section “Statistics” for more information). This approach has been found to be promising (Carpenter and Kenward, 2008; Sterne et al., 2009), however, some pitfalls have been outlined (Carpenter and Kenward, 2008; Sterne et al., 2009). With regard to the current study, these pitfalls specifically concern the small number of observations, limiting the modelling of the unobserved data distribution as well as issues with data normality before multiple imputations. We dealt with those pitfalls by reducing the percentage of missing data via an averaging procedure (i.e., a mean of night 1 and 10 was calculated) and applying the fully conditional specification imputation method (Carpenter and Kenward, 2008). These two interventions meant that (1) fewer missing data were present and thus needed to be estimated based on a small number of observations and (2) the appropriate conditional model (i.e., linear regression) was used to impute data for each variable (Carpenter and Kenward, 2008). Despite these two interventions, the authors of course recognise that the missing data issue is a limitation of the current study. Moreover, we are aware that opting for the last-observation-carried-forward-method in the averaging procedure produces bias (Kenward and Molenberghs, 2009). To minimize the impact of the possibly induced bias on the interpretation of our results, it was decided not to focus on the effect of time in the statistical analysis that is described in section “Statistics Focusing on a Possible Interaction Between Hypoxia and BR.” Rather we focused on the effects of PO_2_ and activity.

The timing of the baseline PSG-recording differed between participants (see section “Sleep Monitoring” for more information). An analysis (see section “Statistics”) was performed to evaluate whether timing of the baseline PSG-recording influenced the sleep characteristics (i.e., a first night-effect). This analysis eventually demonstrated that only three of the 30 PSG-outcomes differed between the four participants that performed their baseline PSG-recording before their first condition and the other eight participants that performed it before their second or third condition. These differences could be related to the first night-effect, but, due to the small sample size (4 vs 8 participants) it is impossible to rule out a type I error (i.e., false positive results). Moreover, because only three of the thirty PSG-outcomes significantly differed between both groups of participants, the supplemental analysis appears to confirm that, in case of a first night-effect, this only minorly impacted the polysomnographic data. In addition, the randomisation of both the conditions (i.e., NBR, HBR, and HAMB) and the baseline night, resulted in the possibility to filter out a first night-effect when a two-way repeated-measures ANOVA is performed (see section “Statistics Focusing on a Possible Interaction Between Hypoxia and BR”).

## Conclusion

A 10-day exposure to hypoxia and BR negatively impacted sleep on multiple levels as in macrostructure, microstructure and respiratory functioning. Hypoxic exposure (i.e., HBR and HAMB) was found to negatively impact multiple aspects of sleep (i.e., sleep maintenance and efficiency, sleep architecture, sleep fragmentation, and sleep-related respiration), while BR (i.e., NBR and HBR) specifically impaired sleep maintenance and efficiency. In terms of the percentage amount of N3 sleep (N3%), a significant interaction between PO_2_ and activity was found. Hypoxia appeared to have less adverse effects on the participants N3% when inactive (NBR vs HBR; –3.8%) compared to when active (pNAMB vs HAMB; –10.2%). In comparison to an identical male study (i.e., LunHab), some sex-specific differences in the acclimatisation process to BR and hypoxia appear to exist. Women did not acclimate to either hypoxia or BR in terms of sleep architecture or WASO, while the men did (Rojc et al., 2014). In the data set that was analysed and interpreted in the present study, data were missing to some extent (i.e., 20.8%). Therefore, multiple imputation was used, and our results should be considered as exploratory. To further progress our understanding of the underlying mechanisms of the hypoxia- and BR-related deteriorations in sleep, future research should focus on confirming the findings that we observed by analysing the imputed data set and assessing the stability of the interindividual and sex-specific differences in the effect of hypoxia and BR on sleep.

## Data Availability Statement

The raw data supporting the conclusions of this article will be made available by the authors, without undue reservation.

## Ethics Statement

The studies involving human participants were reviewed and approved by the National Committee for Medical Ethics at the Ministry of Health of the Republic of Slovenia. The patients/participants provided their written informed consent to participate in this study.

## Author Contributions

HF and AM performed the material preparation and data collection. JV, NP, OM, BD, AM, and IM performed the statistical analysis. JV and NP wrote the first draft of the manuscript and all authors commented on previous versions of the manuscript. All authors developed the study conception and design via open discussion, read and approved the final manuscript.

## Conflict of Interest

The authors declare that the research was conducted in the absence of any commercial or financial relationships that could be construed as a potential conflict of interest.

## Publisher’s Note

All claims expressed in this article are solely those of the authors and do not necessarily represent those of their affiliated organizations, or those of the publisher, the editors and the reviewers. Any product that may be evaluated in this article, or claim that may be made by its manufacturer, is not guaranteed or endorsed by the publisher.
