# Supplementary material for: Adult Female Sleep During Hypoxic Bed Rest
Source: Front Neurosci. 2022 May 10;16:852741. doi: 10.3389/fnins.2022.852741 (PMC9127600; doi:10.3389/fnins.2022.852741)
Supplement: Supplementary file 1 [file Table_1.docx]

| **Supplemental Table 1.** Comparison of all polysomnography-related data between participants with complete (i.e., COMP; n=7) and incomplete data (i.e., INCOMP; n=5). Mean (M) and standard deviation (SD) are depicted for each group of participants, independently from condition. see *2.6 Statistics* for more information. | | | | |
| --- | --- | --- | --- | --- |
|  | COMP |  | INCOMP |  |
|  | *M* | *SD* | *M* | *SD* |
| *Sleep maintenance and efficiency* |  |  |  |  |
| TST (min) | 395 | 52 | 354 | 80 |
| SOL (min) | 16 | 13 | 38 | 27 |
| WASO (min) | 39 | 24 | 44 | 34 |
| EMA (min) | 30 | 19 | 31 | 19 |
| SE (%) * | 82 | 6 | 77 | 14 |
| *Sleep architecture* |  |  |  |  |
| N1 (% of TST) * | 15.4 | 9.9 | 14.3 | 7.6 |
| N1 (min) | 60 | 40 | 50 | 28 |
| N2 (% of TST) | 46.2 | 9.0 | 49.7 | 10.2 |
| N2 (min) | 184 | 44 | 175 | 57 |
| N3 (% of TST) | 24.9 | 7.4 | 22.0 | 10.3 |
| N3 (min) | 97 | 26 | 76 | 34 |
| REM (% of TST) | 13.5 | 5.1 | 14.0 | 10.0 |
| REM (min) | 55 | 22 | 53 | 41 |
| REM latency (min) | 165 | 55 | 146 | 100 |
| N3 latency (min) | 24 | 18 | 27 | 14 |
| Alpha/delta sleep (# of intrusions) | 28 | 27 | 24 | 11 |
| *Sleep fragmentation* |  |  |  |  |
| ArI (# of events/h of TST) | 20.9 | 16.0 | 28.6 | 19.5 |
| Arousals (# of events) | 138 | 109 | 162 | 91 |
| RERA (# of events) | 105 | 101 | 133 | 82 |
| MRA (# of events) | 10 | 6 | 9 | 7 |
| SAR (# of events) | 24 | 13 | 20 | 15 |
| *Sleep-related respiration* |  |  |  |  |
| AHI (# of events/h of TST) | 22.6 | 41.8 | 22.2 | 20.1 |
| HI (# of events/h of TST) | 4.7 | 4.0 | 15.3 | 16.1 |
| OAI (# of events/h of TST) * | 1.1 | 1.0 | 1.6 | 1.4 |
| CAI (# of events/h of TST) | 16.7 | 39.1 | 5.3 | 5.2 |
| MAI (# of events/h of TST) | 0.1 | 0.2 | 0.0 | 0.0 |
| RDI (# of events/h of TST) | 31.1 | 41.4 | 37.5 | 27.1 |
| ODI (# of events/h of TST) | 23.1 | 42.6 | 21.1 | 21.8 |
| MinSpO_2_% | 82 | 10 | 82 | 7.6 |
| *Periodic leg movements* |  |  |  |  |
| PLMSI (# of events/h of TST) | 1.0 | 2.0 | 0.7 | 1.6 |
| Legend: TST=total sleep time; SOL=sleep onset latency; WASO=wake after sleep onset; EMA=early morning awakening (i.e., wakefulness after sleep offset); REM=rapid eye movement sleep; N1%=percentage amount N1 of TST; N2%=percentage amount N2 of TST; N3%=percentage amount N3 of TST; REM%=percentage amount REM of TST; Alpha/delta sleep=number of intrusions of alpha activity during SWS; ArI=arsousal index; Arousals=total number of arousals; RERA=respiratory-related arousals; MRA=movement-related arousals; SAR=spontaneous arousals; AHI=apnea-hypopnea index; HI=hypopnea index; OAI=obstructive apnea index; CAI=central apnea index; MAI=mixed apnea index; RDI=respiratory disturbance index (RDI=AHI+[RERA/h]); ODI=oxygen desaturation index (ODI=number>3% drops in SpO_2_/h); MinSpO_2_%=minimal blood oxygen saturation; PLMSI=periodic leg movement during sleep index | | | | |
