# Supplementary material for: Adult Female Sleep During Hypoxic Bed Rest
Source: Front Neurosci. 2022 May 10;16:852741. doi: 10.3389/fnins.2022.852741 (PMC9127600; doi:10.3389/fnins.2022.852741)
Supplement: Supplementary file 2 [file Table_2.docx]

| **Supplemental Table 2.** Overview of all polysomnography-related data for the four participants that performed the baseline PSG-recording in the BDC phase before their first condition (i.e., pNAMB_1) and for the eight participants that performed the baseline PSG-recording in the BDC phase before their second or third condition (i.e., pNAMB_2). Mean (M) and standard deviation (SD) are depicted for the data set in which missing data was imputed (i.e., in pNAMB_1, n=4 for each outcome; in pNAMB_2, n=8 for each outcome; see *2.6 Statistics* for more information). | | | | |
| --- | --- | --- | --- | --- |
|  | pNAMB_1 |  | pNAMB_2 |  |
|  | *M* | *SD* | *M* | *SD* |
| *Sleep maintenance and efficiency* |  |  |  |  |
| TST (min) | 387 | 20 | 362 | 104 |
| SOL (min) | 10 | 10 | 15 | 8 |
| WASO (min) | 57 | 19 | 52 | 53 |
| EMA (min) | 14 | 10 | 12 | 9 |
| SE (%) * | 83 | 4 | 89 | 3 |
| *Sleep architecture* |  |  |  |  |
| N1 (% of TST) * | 14.6 | 2.8 | 10.1 | 3.1 |
| N1 (min) | 57 | 12 | 41 | 20 |
| N2 (% of TST) | 46.9 | 10.8 | 41.4 | 5.8 |
| N2 (min) | 181 | 41 | 166 | 53 |
| N3 (% of TST) | 24.8 | 6.1 | 31.8 | 9.0 |
| N3 (min) | 96 | 22 | 104 | 18 |
| REM (% of TST) | 13.8 | 3.9 | 11.3 | 8.6 |
| REM (min) | 54 | 18 | 51 | 40 |
| REM latency (min) | 162 | 51 | 150 | 71 |
| N3 latency (min) | 21 | 12 | 21 | 9 |
| Alpha/delta sleep (# of intrusions) | 13 | 8 | 30 | 23 |
| *Sleep fragmentation* |  |  |  |  |
| ArI (# of events/h of TST) | 11.4 | 4.4 | 11.3 | 2.9 |
| Arousals (# of events) | 73 | 28 | 63 | 26 |
| RERA (# of events) | 45 | 28 | 37 | 17 |
| MRA (# of events) | 7 | 7 | 13 | 5 |
| SAR (# of events) | 22 | 6 | 23 | 13 |
| *Sleep-related respiration* |  |  |  |  |
| AHI (# of events/h of TST) | 3.1 | 2.1 | 3.7 | 1.8 |
| HI (# of events/h of TST) | 2.3 | 1.7 | 6.8 | 9.0 |
| OAI (# of events/h of TST) * | 0.4 | 0.7 | 1.6 | 1.0 |
| CAI (# of events/h of TST) | 0.4 | 0.2 | 1.3 | 0.8 |
| MAI (# of events/h of TST) | 0 | 0 | 0 | 0 |
| RDI (# of events/h of TST) | 9.0 | 4.6 | 9.1 | 2.6 |
| ODI (# of events/h of TST) | 0.9 | 0.3 | 4.2 | 2.4 |
| MinSpO_2_% | 90 | 1 | 90 | 2 |
| *Periodic leg movements* |  |  |  |  |
| PLMSI (# of events/h of TST) | 0.2 | 0.3 | 0.2 | 0.2 |
| Legend: * indicates a significant difference between pNAMB_1 and pNAMB_2; TST=total sleep time; SOL=sleep onset latency; WASO=wake after sleep onset; EMA=early morning awakening (i.e., wakefulness after sleep offset); REM=rapid eye movement sleep; N1%=percentage amount N1 of TST; N2%=percentage amount N2 of TST; N3%=percentage amount N3 of TST; REM%=percentage amount REM of TST; Alpha/delta sleep=number of intrusions of alpha activity during SWS; ArI=arsousal index; Arousals=total number of arousals; RERA=respiratory-related arousals; MRA=movement-related arousals; SAR=spontaneous arousals; AHI=apnea-hypopnea index; HI=hypopnea index; OAI=obstructive apnea index; CAI=central apnea index; MAI=mixed apnea index; RDI=respiratory disturbance index (RDI=AHI+[RERA/h]); ODI=oxygen desaturation index (ODI=number>3% drops in SpO_2_/h); MinSpO_2_%=minimal blood oxygen saturation; PLMSI=periodic leg movement during sleep index | | | | |
